# Microbiota-Associated Biofilm Regulation Leads to *Vibrio cholerae* Resistance Against Intestinal Environmental Stress

**DOI:** 10.3389/fcimb.2022.861677

**Published:** 2022-04-27

**Authors:** Jennifer Y. Cho, Rui Liu, Ansel Hsiao

**Affiliations:** ^1^Department of Microbiology and Plant Pathology, University of California, Riverside, Riverside, CA, United States; ^2^Department of Biochemistry, University of California, Riverside, Riverside, CA, United States; ^3^Graduate Program in Genetics, Genomics, and Bioinformatics, University of California, Riverside, Riverside, CA, United States

**Keywords:** biofilm, *Vibrio cholerae*, pathogenesis, microbiota, bile acids, reactive oxygen species

## Abstract

The commensal microbes of the gut microbiota make important contributions to host defense against gastrointestinal pathogens, including *Vibrio cholerae*, the etiologic agent of cholera. As interindividual microbiota variation drives individual differences in infection susceptibility, we examined both host and *V. cholerae* gene expression during infection of suckling mice transplanted with different model human commensal communities, including an infection-susceptible configuration representing communities damaged by recurrent diarrhea and malnutrition in cholera endemic areas and a representative infection-resistant microbiota characteristic of healthy individuals. In comparison to colonization of animals with resistant microbiota, animals bearing susceptible microbiota challenged with *V. cholerae* downregulate genes associated with generation of reactive oxygen/nitrogen stress, while *V. cholerae* in these animals upregulates biofilm-associated genes. We show that *V. cholerae* in susceptible microbe infection contexts are more resistant to oxidative stress and inhibitory bile metabolites generated by the action of commensal microbes and that both phenotypes are dependent on biofilm-associated genes, including *vpsL*. We also show that susceptible and infection-resistant microbes drive different bile acid compositions *in vivo* by the action of bile salt hydrolase enzymes. Taken together, these findings provide a better understanding of how the microbiota uses multiple mechanisms to modulate the infection-associated host environment encountered by *V. cholerae*, leading to commensal-dependent differences in infection susceptibility.

## Introduction

In the transition from the aquatic reservoir into the human gastrointestinal tract, *Vibrio cholerae* encounters multiple host defense mechanisms, including low pH, oxidative stress, and bile acids (Louis and O’byrne, 2010). Growing evidence indicates that the resident community of gut microbes, the gut microbiota, is also an essential factor affecting host susceptibility to *V. cholerae* infection, using multiple mechanisms to modulate pathogen fitness and gene expression during infection (Hsiao et al., 2014; Alavi et al., 2020; Cho et al., 2021). The gut microbiota is highly complex and varies dramatically between individuals, leading to individual-specific effects on *V. cholerae* susceptibility driven partially by community-specific effects on *V. cholerae* behavior during infection (Hsiao et al., 2014; Alavi et al., 2020). In cholera-endemic areas, environmental insults such as malnutrition and diarrhea of multiple etiologies drive some individuals’ gut communities to recurring damaged or dysbiotic states that are unable to resist the colonization of *V. cholerae* (Hsiao et al., 2014; David et al., 2015; Alavi et al., 2020). Commensal microbes are able to modulate *V. cholerae* fitness and behavior during infection through numerous mechanisms including quorum sensing regulation of virulence genes (Hsiao et al., 2014) and direct competition *via* the type VI secretion system (T6SS) (Zhao et al., 2018). In addition to direct competition and modulating *V. cholerae* transcription during infection, the gut microbiota is also able to broadly modulate gut environmental conditions, such as through manipulation of oxidative conditions as well as host metabolites such as bile. Both of these factors are active during *V. cholerae* infection; transcriptomic studies of cholera patients reveal an increase in expression of host enzymes responsible for oxidative stress (Ellis et al., 2015; Bourque et al., 2018), and microbiota-dependent modulation of bile acid levels has been shown to affect *V. cholerae* virulence gene expression (Alavi et al., 2020).

A key adaptation of *V. cholerae* to environmental stresses is the ability to form biofilms, which in addition to protecting against acid, predation in the aquatic reservoir, and bacteriostatic molecules in the host gut such as bile also modulates *V. cholerae* infectivity and epidemic spread (Zhu and Mekalanos, 2003; Matz et al., 2005; Hung et al., 2006; Hay and Zhu, 2015). Here, we show using model human microbiota compositions that commensals are able to differentially affect *V. cholerae* fitness by modulating the expression of biofilm-associated genes in *V. cholerae*. This process intersects with the microbiota-specific manipulation of gut redox conditions and bile acid composition that both inhibit *V. cholerae* fitness in a biofilm-dependent fashion. Our findings suggest that the composition and biochemical functions of the gut microbiota are able to use reinforcing mechanisms, leading to commensal-dependent infection-permissive and restrictive conditions in the mammalian gastrointestinal tract.

## Methods

### Bacterial Strains and Culture Condition

All *V. cholerae* strains were derived from the C6706 O1 El Tor pandemic strain and grown in LB liquid media with appropriate antibiotics at 37°C with agitation. All anaerobic strains were grown in LYHBHI liquid media (BHI supplemented to 5 g/l yeast extract, 5 mg/l hemin, 1 mg/ml cellobiose, 1 mg/ml maltose, and 0.5 mg/ml cysteine–HCl) at 37°C in a Coy chamber under anaerobic conditions (5% H_2_, 20% CO_2_, balance N_2_) (Alavi et al., 2020). All anaerobic strains used in animal experiments were grown for 48 h, then 1:100 subcultured into fresh media and grown an additional 48 h prior to gavage. *vpsL* mutants were constructed using natural transformation based on the published method (Dalia et al., 2014). Approximately a 3-kb *vpsL* upstream (forward primer: 5′-GTTAAGAGCACCGATTGCAC-3′, reverse primer: 5′-GTCGACGGATCCCCGGAATCTTCATCACTAGACGCTCCTAAC-3′) and downstream (forward primer: 5′-GAAGCAGCTCCAGCCTACAGCGTATTAAGACAGGGCACTTG-3′, reverse primer: 5′-GCATTTTTTACCGTCAGGGTC-3′) sequence was amplified and added to ends of a trimethoprim resistance (Tm^R^) cassette amplified from SAD530 using primers ABD123 (5′-ATTCCGGGGATCCGTCGA-3′) and ABD124 (5′-TGTAGGCTGGAGCTGCTTC-3′) by overlapping PCR using Phusion High-Fidelity PCR Master Mix (Thermo Fisher Scientific, Waltham, MA, USA). The first round of PCR consisted of an equimolar *vpsL* upstream fragment, a *vpsL* downstream fragment, and Tm^R^ cassette amplicons with a cycle condition of 98°C for 30 s, followed by 15 cycles (94°C for 10 s, 50°C for 30 s, 72°C for 4 min), and 72°C for 10 min. A *vpsL* upstream forward primer and a downstream reverse primer were added to the PCR reaction which was then continued using cycle conditions as described above. The final PCR product was added to 0.45 ml of instant ocean sea salt solution (7 mg/ml) with chitin-induced *V. cholerae* and incubated for 16–24 h at 30°C stationary, allowing natural competence. 0.5 ml LB was then added, and the mixture was incubated for 2–3 h at 30°C with agitation. The final mixture was then plated on an LB agar plate with 10 µg/ml trimethoprim for overnight incubation at 30°C to select for transformants. The final picked mutants were checked by Sanger sequencing.

### Animal Experiments

Specific pathogen-free (SPF) CD-1 suckling animals were purchased from Charles River Laboratories. Upon receipt, suckling mice were fasted, and an equivalent of about 1 mg/g body weight streptomycin was gavaged with 30-gauge plastic tubing. Pups were then placed with a lactating dam for 1 day before being inoculated with communities and *V. cholerae*. Human gut bacterial strains were prepared by taking a total 300 µl of OD_600_ = 0.4 equivalent culture per animal divided evenly across constituent commensal strains. Appropriate amounts of anaerobic strain culture were pooled and pelleted by centrifugation and resuspended in 25 µl of fresh LYHBHI per animal. Mice then received 25 µl of the community mixture and 25 µl of *V. cholerae* (1 × 10^4^–1 × 10^5^ CFU) in PBS (Alavi et al., 2020). 4-day-old suckling CD-1 mice were fasted for 1.5 h prior to all gavages. 18 hours after infection, pups were sacrificed, intestinal tissue was homogenized in 5 ml of PBS, and *V. cholerae* was plated on a streptomycin LB-agar plate for enumeration and fitness assays. For RNA assays, 1 ml of intestinal homogenate from each infected pup was centrifuged at 14,000 rpm, the supernatant was removed, and the pellet was resuspended in 500 µl of TRIzol (Ambion, Foster City, CA, USA) at -80°C.

### Quantitative PCR Analysis of Model Microbiome

DNA was extracted from an intestinal homogenate and used as template for model strain detection by quantitative PCR. 500 µl of intestinal homogenate was mixed with 210 µl of 20% SDS, 500 µl phenol:chloroform:isoamyl-alcohol (24:24:1, Fisher Scientific), and 500 µl of 0.1-mm zirconia beads (BioSpec, Bartlesville, OK, USA). The mixture was then lysed with a bead beater at 2,400 RPM for 3 min. The qPCR assay to detect model strains was performed using a universal 16S rRNA gene primer set to detect total bacterial load (forward primer: 5′-GTGSTGCAYGGYTGTCGTCA-3′, reverse primer: 5′-ACGTCRTCCMCACCTTCCTC-3′) (Horz et al., 2005). Each reaction was done in triplicates with 12.5 µl of iQ SYBR Green Supermix (BIO-RAD2), 1 µl of 10µM forward and reverse primers, 5.5 µl nuclease-free water, and 5 µl of extracted DNA (200 ng/µl).

### RNA Extraction, RNA-Seq Library Prep, and Sequencing

Total RNA was extracted using TRIzol according to the manufacturer’s instructions. DNA was degraded using Baseline-ZERO DNase (Lucigen, Middleton, WI, USA), and the samples were then treated with TRIzol again for purification. A sequencing library was prepared using the Ovation Mouse RNA-Seq System with addition of *V. cholerae* enrichment primers (NuGEN, San Carlos, CA, USA). Final libraries were checked using an Agilent Bioanalyzer, and a 75-bp single-read sequencing was performed on Illumina NextSeq500.

### RNA-Seq Analysis

All RNA-Seq-sequenced libraries were trimmed using Trimmomatic v0.39 (Bolger et al., 2014), and reads were aligned to mouse and *V. cholerae* genomes, using HISAT2 version 2.1.0 (Kim et al., 2015) with reference genomes GCF_000001635.26_GRCm38.p6 and GCF_000006745.1_ASM674v1. HTSeq (Anders et al., 2015) was used to calculate raw counts. After rRNA and tRNA were removed, differential expression analysis was conducted using edgeR (Robinson et al., 2009; Mccarthy et al., 2012). Statistical analysis and visualization of *V. cholerae* pathway profiles were performed using package clusterProfiler (Yu et al., 2012; Wu et al., 2021) in R with the selected database KEGG, and genes with FDR <0.05 and FC >1 or FC <-1 were used for further analyses.

### Measurement of H_2_O_2_ Levels in Colonized Intestines

Intestinal homogenate H_2_O_2_ levels were measured using the Amplex UltraRed reagent kit (Invitrogen, Carlsbad, CA, USA). 50 µl of intestinal homogenate was collected and mixed with 50 µl of working solution (5 mM Amplex UltraRed reagent, 10 U horseradish peroxidase, HRP, 0.05 M sodium phosphate, pH 7.4) immediately after homogenization. The mixture was incubated at room temperature for 30 min, and absorbance was measured (560 nm). The H_2_O_2_ level was then determined based on the H_2_O_2_ standard curve.

### *V. cholerae Ex Vivo* Oxidative Stress Resistance Assays

50 µl of intestinal tissue homogenates collected from *V. cholerae*-infected suckling mice colonized with different commensal groups was incubated with 1 mM of hydrogen peroxide for 1 h at room temperature. After incubation, 10 µl of untreated and treated homogenates was collected, serially diluted, and plated on selective agar to determine survival rates.

### Intestinal Homogenate *Ex Vivo* Treatment With Pure Culture

Intestinal homogenates were prepared as described in previously published methods (Alavi et al., 2020). Briefly, intestines were collected from 6-day-old CD-1 suckling mice fasted for 18 h and homogenized in 2.5 ml sterile water, homogenates were centrifuged, and then aqueous supernatants were collected. The supernatant was treated at 100℃ for 30 min and filter sterilized with a 0.22-µM filter. The sample was then dried with the Savant Integrated SpeedVac System (Fisher Scientific) and resuspended in 0.5 ml sterile water. 2 ml of acetonitrile (Sigma-Aldrich, St. Louis, MO, USA) was added to the sample and incubated at room temperature for 20 min to deproteinize after vortexing (Humbert et al., 2012). The aqueous layer was then filter sterilized, dried, and resuspended in 0.5 ml sterile water. 1.5 ml of OD_600_ = 0.4 pure culture was pelleted and resuspended in purified homogenates then incubated anaerobically for 24 h at 37°C. The culture was heat-treated at 100℃ for 30 min then centrifuged. The aqueous layer was then filter-sterilized with a 0.22-µM filter after being cooled to room temperature and sent for mass spectrometry analysis.

### Quantitative PCR Analysis of VPS Gene Expression

An overnight culture of *V. cholerae* was 1:100 inoculated into fresh LB liquid media containing 100 µg/ml streptomycin and grown to OD_600_ = 0.3. 50 µM of H_2_O_2_ or an equal volume of PBS was added to the culture and incubated for 2 h at 37°C with shaking. The cultures were collected, and RNA was extracted using TRIzol according to the manufacturer’s instructions. 2 µg of total RNA was then used for reverse transcription PCR using the SuperScript™ IV One-Step RT-PCR System (Invitrogen). qPCR assays were preformed using gene-specific primer sets targeting *vpsR* (forward primer: 5′-TGGCACACTGCTGCTAAA-3′, reverse primer: 5′-ACATCGACTGCACGAACC-3′), *vpsT* (forward primer: 5′-ACCCTGATCAAAGGCATGAG-3′, reverse primer: 5′-GTGAGGTCACGACTGAGTTAC-3′), and *vpsL* (forward primer: 5′-CAGTATGCGAGTGATGGATAATGG-3′, reverse primer: 5′-TCGTGGATCGCCTTTGGT-3′), with *recA* (forward primer: 5′-ATTGAAGGCGAAATGGGCGATAG-3′, reverse primer: 5′-TACACATACAGTTGGATTGCTTGAGG-3′) as the reference gene. Each reaction was done in triplicate with 12.5 µl of iQ SYBR Green Supermix (BIO-RAD2), 1 µl of 10 µM forward and reverse primers each, 8.5 µl nuclease-free water, and 2 µl of 2-fold-diluted RT-PCR reaction product.

### Measurement of Bile Acid Processing by *B. obeum* BSH

Overnight cultures of *E. coli bsh^c^* and *E. coli* vector with pZE21 (Alavi et al., 2020) were 1:100 inoculated into fresh LB liquid media containing 50 µg/ml kanamycin and incubated for 24 h at 37℃ with shaking. The cultures were normalized to 1.5 ml of OD_600_ = 0.4 culture, pelleted, and washed with 1.5 ml of PBS. The pellet was resuspended in 1.5 ml of PBS containing 2 mM of taurodeoxycholic acid (TDCA) and transferred to glass tubes then incubated at 37°C stationary for 24 h. After the incubation, supernatants were collected by centrifuge and filter-sterilized with 0.22-µM filters. Collected supernatants were sent for mass spectrometry analysis and tested for *V. cholerae* growth inhibition.

### *V. cholerae* Growth Curve Measurements

Supernatants collected as previously described were 1:1 mixed with a 1:1,000 diluted log-phase *V. cholerae* culture in a 96-well plate. OD_600_ was then taken every 30 min for 20 h with a BioTek Synergy HTX multi-mode reader under 37°C with orbital shaking at 180 cpm (6 mm) using Gen5 2.09.

### Bile Acid Extraction and Preparation From *In Vitro* Enzymatic Assays

Bile acids were extracted from *in vitro* enzymatic assays for LC-MS measurements. An aliquot of 200 µl of the assay was acidified to a final concentration of 2% acetic acid. A liquid–liquid extraction was performed with 300 µl of ethyl acetate. The supernatant was collected (200 µl) and dried down under a stream of nitrogen. The tube content after drying was resuspended in 1 ml methanol and diluted 20× for LC-MS analysis.

### Liquid Chromatography Mass Spectrometry Measurement of Bile Salts

Standards for TDCA and deoxycholic acid (DCA) (1 µM) were used to determine retention times and fragmentation patterns for each compound. Chromatographic separations were conducted in a Waters I-Class UPLC System using an ACQUITY UPLC BEH C18 column (2.1 × 100 mm, 1.7 µM; Waters, Milford, MA, USA). Water with 0.1% formic acid (A) and acetonitrile with 0.1% formic acid (B) were used as mobile phases. Sample separation was conducted after 1-µl injections with the column kept at 40°C, with a flow rate of 0.4 ml/min under the following gradient: 0 min, 10% B; 1 min, 10% B; 1.5 min, 25% B; 8 min, 45% B, 10 min, 90% B; 16 min, 90% B, 17 min, 45% B, 19 min, 25% B; 21 min, 10% B; 26 min, 10% B. Mass spectrometry measurements were conducted in a SYNAPT G2-Si quadrupole time-of-flight mass spectrometer (Waters). The instrument was operated in negative ion mode under MS/MS conditions with a source capillary voltage of 2.5 kV, source temperature of 150°C, desolvation temperature of 600°C, and desolvation gas at 600 l/h. Leucine enkephalin was used as lock spray standard for mass correction. The following mass transitions (m/z) produced by the indicated collision energies (trap and transfer) were used for signal identification of TDCA and DCA [TDCA, 79.95 and 124.00 (CE 40 V); DCA, 343.26 and 391.28 (CE 25 V)]. The QuanLynx software (Waters) was used for peak integration and peak area calculation and processing. A quality control sample was produced by pooling equal amounts of samples and used to monitor system stability and reproducibility.

### Bile Acid Growth Inhibition of Biofilm-Associated *V. cholerae*

Overnight *V. cholerae* cultures were 1:100 inoculated into fresh LB liquid media with 100 µg/ml streptomycin and 2 ml of dilution aliquoted into 100 × 15-mm sterilized polystyrene petri dishes with 10-mg glass beads (50–100 µm, Polysciences, Warrington, PA, USA), followed by incubation for 24 h at room temperature with slow shaking (10 rpm) (Zhu and Mekalanos, 2003). Planktonic cells were collected by pipetting media, followed by pelleting *via* centrifugation and resuspension in LB media containing 1 mM of different bile acids (diluted from 100× stock solution in dimethyl sulfoxide, DMSO). Beads were rinsed with 1× PBS to remove residual planktonic cells, and the biofilm-associated bacteria on the beads were then treated with 5 ml LB liquid media containing 1 mM of different bile acids. For both treatments, an identical volume of DMSO as bile acid stock was added to LB liquid media as control. Both planktonic cells and biofilm on the beads were treated for 4 h, and surviving bacteria were then enumerated by serial dilution and plating.

### Statistical Analysis

Statistical analysis was performed using GraphPad Prism 9 (GraphPad Software, Inc., La Jolla, CA, USA). Student’s unpaired *t-*test was used to analyze *in vitro* and *ex vivo* CFU and ratio group differences. The Mann–Whitney *U-*test was used to analyze *in vivo* CFU level differences between groups. Comparisons where P < 0.05 were considered statistically significant. Results are representative of independent repeated experiments.

## Results

### Bacterial Community-Dependent *V. cholerae* Colonization

**Figure 1:**
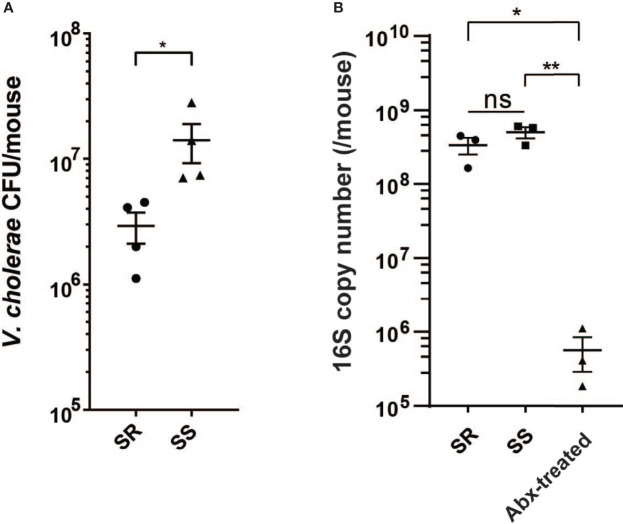
V*. cholerae* colonization and fitness varies with model commensal microbiota. **(A)** *V. cholerae* colonization of antibiotic-cleared 5-day-old suckling CD-1 mice after co-gavage with model microbial mixtures. **(B)** Bacterial 16S copy number in intestinal homogenates (n = 3–4, *P value < 0.05, **P value < 0.01, ns, not significant).

Previous studies have shown that specific human gut microbiota compositions can affect *V. cholerae* colonization using the antibiotic-cleared infant CD-1 suckling mouse model, with dysbiotic gut microbiota being more susceptible to infection compared to prototypical microbial communities characteristic of healthy individuals (Alavi et al., 2020). As the gut microbiota is highly diverse from individual to individual, we used simplified model groups of microbes representative of “healthy” and “unhealthy” or dysbiotic states to examine the effects of microbiota structure on *V. cholerae* behavior during infection. The simplified infection-resistant model community (SR) consists of the species representing the major taxa found in either the healthy individuals in Bangladesh (Hsiao et al., 2014), *Blautia obeum* (previously classified as *Ruminococcus obeum*), *Bacteroides vulgatus*, and *Clostridium scindens*. The low-diversity dysbiotic state induced by recurrent diarrhea or malnutrition was represented by *Streptococcus salivarius* subsp. *salivarius* (SS). 4-day-old suckling mice were first treated with streptomycin to remove the host microbiome then coinfected with *V. cholerae* and the community strains after 24 h. The intestine was collected 18 h postinfection and homogenized for CFU enumeration of *V. cholerae*, with aliquots of homogenates also stored for host and bacterial RNA-Seq analysis. In accordance with previous findings using healthy and dysbiotic human gut microbiota (Alavi et al., 2020), we repeatedly observed that *V. cholerae* was able to colonize SS-containing animals at around 10^7^ CFU; when coinfected with SR, *V. cholerae* colonization was around ~4-fold lower (**Figure 1A**). We also verified the colonization of model microbes by measuring total bacterial load using qPCR assay with universal 16S primers after antibiotic treatment and after gavage; total bacterial 16S copy number was significantly higher after bacterial gavage compared to antibiotic treated mice (**Figure 1B**). These findings replicate previous studies that these model microbes are capable of colonizing mice intestine (Alavi et al., 2020).

### RNA-Seq Analysis Reveals Microbiota-Dependent Expression of Biofilm-Associated Genes

**Figure 2:**
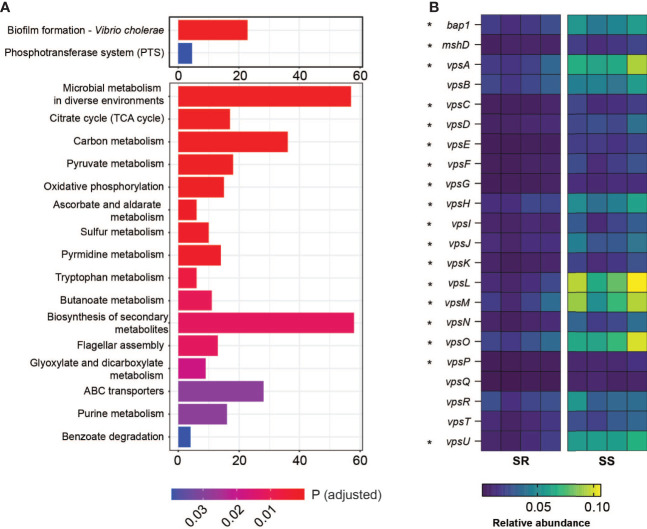
Differential gene regulation in *V. cholerae* on coinfection with different model communities. **(A)** Number of genes involved in indicated KEGG gene pathways relatively significantly upregulated in *V. cholerae* coinfected with SS vs. SR (upper panel) and SR vs. SS (bottom panel). **(B)** Relative abundance (%) of genes involved in *Vibrio* polysaccharide formation and attachment from *V. cholerae* coinfected with SR or SS. Starred genes (*) are significantly differentially expressed across microbiota groups (*P < 0.005).

Alavi et al. determined that one contributor of microbiota-dependent variation in infection resistance was a variable level of commensal-encoded bile salt hydrolase (BSH) that de-conjugates the attached amino acid of the bile acid taurocholic acid (TCA), leading to differences in induction of virulence gene expression; *B. obeum* (an SR organism) was a key BSH producer, while *S. salivarius* lacked BSH activity (Alavi et al., 2020). To examine other potential contributors to SR/SS infection differences, we examined *V. cholerae* and host gene expression during infection of suckling mice. We extracted RNA from intestinal homogenates of animals colonized with both communities and infected with *V. cholerae* and used RNA-Seq to examine community-dependent *V. cholerae* gene expression patterns. Using pathway analysis with the Kyoto Encyclopedia of Genes and Genomes (KEGG) (Kanehisa and Goto, 2000; Kanehisa, 2019; Kanehisa et al., 2021), we found that *V. cholerae* coinfected with SS significantly upregulated biofilm formation and phosphotransferase system pathways relative to infection of SR mice; in SR mice, 16 pathways were increased in expression relative to SS, predominantly metabolic pathways including carbon metabolism, amino acid metabolism, and oxidative phosphorylation (**Figure 2A**). Biofilm is a population of microbes aggregated and attached to a surface in a matrix of extracellular polysaccharide (EPS), nucleic acid, and proteins. The production and manipulation of *Vibrio* extracellular polysaccharide (VPS) are driven by gene products encoded by two gene clusters *vps*-I (*vpsU*, *vpsA-K*) and *vps*-II (*vpsL*-*Q*) (Yildiz and Schoolnik, 1999; Fong et al., 2010). We observed that the expression of most VPS genes is upregulated 2–4-fold during infection of SS-colonized animals compared to SR-colonized animals. Among VPS genes, *vpsL* displayed the strongest difference in expression between microbiota groups, with 4.5-fold higher in expression in SS vs. SR. VpsL is known to be essential for forming a stable biofilm structure (Fong et al., 2010). Other than the exopolysaccharide matrix production genes, another *vps*-coregulated protein, Bap1 (biofilm associated protein), also displayed a 2-fold increase in expression in infection of SS animals (**Figure 2B**). Bap1 is an extracellular matrix protein that modulates biofilm structure and pellicle formation and mediates cell-to-surface adhesion (Fong and Yildiz, 2008; Yan et al., 2016). Biofilms protect microbes, including *V. cholerae*, from environmental stress including extreme environments and host defense mechanism (Roilides et al., 2015; Yin et al., 2019), and increases infectivity (Zhu and Mekalanos, 2003; Tamayo et al., 2010). Our findings suggest that infection of SS-like permissive, dysbiotic microbiota could induce *V. cholerae* to form a more robust biofilm structure either directly or indirectly, leading to stronger defenses against environmental stress.

### Host iNOS and DUOX Response Toward Microbiome Structure

**Figure 3:**
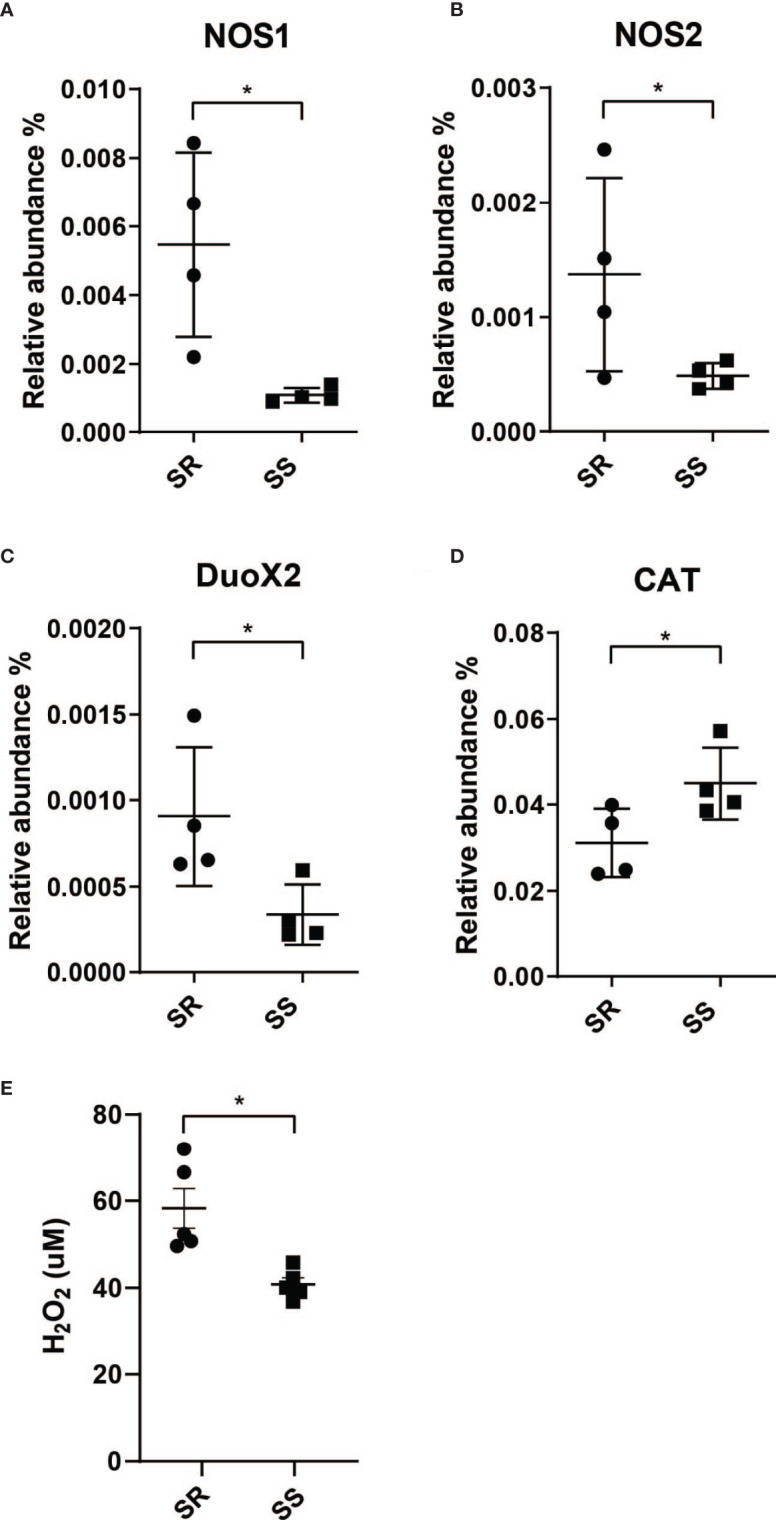
Community-dependent host oxidative stress responses during *V. cholerae* expression. Host genes involved in the synthesis of reactive nitrogen species. **(A)** Nitric oxide synthase 1 (NOS1). **(B)** Nitric oxide synthase 2 (NOS2) and reactive oxygen species. **(C)** Dual oxidase 2 (DUOX2). **(D)** Catalase (CAT). **(E)** H_2_O_2_ level measured from collected intestinal homogenates (n = 4, *P < 0.01).

Given that the SS triggered the expression of the genes associated with biofilm formation in *V. cholerae*, and the critical role of biofilms in defense against environmental stresses, we next examined whether the host environment during infection was modulated by the microbiota through transcriptomic measures of host responses to *V. cholerae* infection in the presence of SR and SS model microbes. We found that mice coinfected with SR and *V. cholerae* exhibited a significantly higher expression of *NOS1*, *NOS2*, and *DUOX2* in the total intestine (**Figures 3A–C**). *NOS2* and *DUOX2* are known to be involved in the production of compounds such as nitric oxide (NO) and extracellular hydrogen peroxide (H_2_O_2_), respectively (Geiszt et al., 2003; El Hassani et al., 2005). In response to numerous gastrointestinal infections, host enzymes are able to generate increased local reactive nitrogen stress (RNS) and reactive oxygen stress (ROS), which collectively form a key mechanism to inhibit pathogens (Paiva and Bozza, 2014). Nitric oxide synthases (NOS) catalyze the production of nitric oxide (NO), which includes three different isoforms, NOS1–3. The NO synthesized by NOS1 and NOS3 mainly acts as a neurotransmitter in the brain and peripheral nervous system and maintains a healthy cardiovascular system (Forstermann and Munzel, 2006; Knott and Bossy-Wetzel, 2009). NOS2 is involved in immune response and NO synthesis. ROS synthesis involves the expression of dual oxidase (DUOX) that catalyzes hydrogen peroxide production (Sommer and Backhed, 2015). Prior studies have demonstrated an increase in both ROS and RNS during *V. cholerae* infection; Ellis et al. showed that cholera patients show higher nitric oxide synthase 2 (NOS2) protein abundant during the acute phase (Ellis et al., 2015), while an examination of cholera patients’ mucosal gene expression by Bourque et al. demonstrated that dual oxidase 2 (*DUOX2*) and *NOS2* are higher in expression during the acute phase (Bourque et al., 2018).

The resulting increase in NO and H_2_O_2_ production leads to high local oxidative stress, which forms a key element of the initial host response toward pathogen infection (Kim and Lee, 2014; Król and Kepinska, 2021). These antimicrobial effects are mediated by several pathways, including conversion of NO to peroxynitrite (OONO^-^), and form OH· and nitrogen dioxide (·NO_2_) (Nathan and Ding, 2010) to cause oxidative damage on phagocytosed pathogens, and pathogen in phagosome, and H_2_O_2_ or through a catalytic reaction to form hydroxyl radicals (OH·) which leads to pathogen DNA breakage, base oxidation and deamination, and lipid peroxidation (Paiva and Bozza, 2014). This increase in ROS production has been observed in cholera patients, as measured by NO metabolite levels in serum and urine (Janoff et al., 1997). Cholera patients’ duodenum samples at the acute phase showed more abundant *NOS2* and *DUOX2* transcript levels compared to the convalescent phase (Ellis et al., 2015; Bourque et al., 2018). Other than higher ROS/RNS enzyme expression, host catalase (*CAT*) gene expression is also induced by infection in the presence of SS (**Figure 3D**). Since catalase is an antioxidant enzyme that promotes the conversion and detoxification of ROS and H_2_O_2_ to prevent oxidative damage to host cells, this further supports an increase in ROS stress during infection of individuals with SR-like microbiota compared to individuals with an SS-like commensal microbe. We then measured H_2_O_2_ production in the homogenates from each model microbiome and found that colonization with the SR community induced significantly higher production of H_2_O_2_ (**Figure 3E**). Given that a significant difference in overall H_2_O_2_ was observed from total organ samples, local differences in levels of H_2_O_2_ at epithelial microenvironments as a function of microbiome and infection may be even higher (Bhattacharyya et al., 2014; Taylor and Colgan, 2017; Burgueno et al., 2019; Imlay, 2019).

### Differential Regulation of Biofilm Formation Leads to Oxidative Stress Resistance

**Figure 4:**
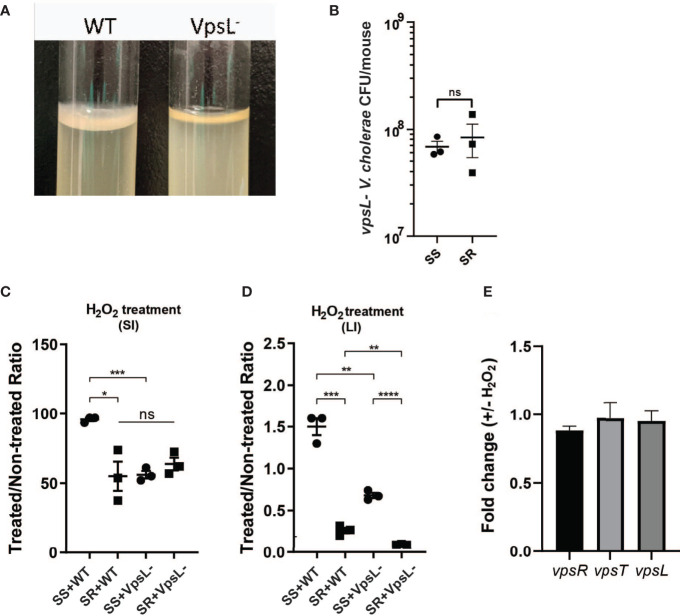
Presence of different commensal microbes during infection drives different biofilm-dependent *V. cholerae* oxidative stress resistances. **(A)** Confirmation of disruption of biofilm formation by *vpsL* gene deletion. **(B)** *vpsL* mutant *V. cholerae* colonization co-gavaged with indicated model microbiota. Survival of *V. cholerae* strains in **(C)** small intestine and **(D)** large intestine of antibiotic-cleared suckling mice co-colonized with indicated commensal communities after hydrogen peroxide treatment. **(E)** qPCR quantification of VPS gene expression *in vitro* after hydrogen peroxide treatment (n = 3, *t* test, *P < 0.05; **P < 0.01; ***P < 0.001; ****P < 0.0001; ns, not significant).

Previous studies have shown that biofilm formation enhances *V. cholerae* resistance toward oxidative stresses driven by early infection (Wang et al., 2018), and our data suggest that SS can induce higher biofilm formation gene expression in *V. cholerae*. To test whether microbiome-dependent differences in biofilm-associated gene expression lead to an increase in oxidative stress resistance, we used an *ex vivo* ROS survival assay. We coinfected suckling animals with wild-type and biofilm defective *vpsL* mutants (**Figure 4A**) and our two model commensal collections. The mutant showed no significant differences in colonization between the two model microbiota (**Figure 4B**). We then isolated *V. cholerae* and associated extracellular structures by homogenizing infected small and large intestinal tissues and then treated *V. cholerae*-containing homogenates with H_2_O_2_. Survival rates of *V. cholerae* were then determined by plating. We found that wild-type *V. cholerae* exhibited >90% H_2_O_2_ survival after infection of the SS-co-colonized small intestine, while *V. cholerae* that colonized SR-bearing animals exhibited only ~55% survival. When the *vpsL* mutant was co-gavaged with the model microbes, this SS-dependent advantage was ablated (**Figure 4C**), suggesting that biofilm formation was necessary for this difference in oxidative stress resistance. We observed the same pattern from the large intestine where the wild-type *V. cholerae* from SS animals exhibited higher levels of *ex vivo* ROS resistance compared to *V. cholerae* from SR animals, and *vpsL* mutants in either microbiota context (**Figure 4D**), although the rate of survival in both colonization contacts was significantly reduced compared to the small intestine, which may indicate that the regulatory effects of commensal microbes are concentrated at the preferred site of colonization of *V. cholerae*, the distal small intestine. To determine whether biofilm biogenesis was induced by hydrogen peroxide, we treated *V. cholerae* culture with H_2_O_2_ *in vitro* and examined *vpsR*, *vpsT*, and *vpsL* expression. Quantitative RT-PCR analysis showed no significant differences in expression level between H_2_O_2_-treated and untreated cultures (**Figure 4E**), suggesting that other mechanisms, potentially microbial in nature, are responsible for observed *in vivo* microbiome-specific differences in *V. cholerae* biofilm production. Taken together, these findings suggest that i) microbiota-associated biofilm expression leads to different levels of biofilm-dependent ROS resistance in *V. cholerae* and ii) that this effect is much more substantial in the preferred site of colonization in the distal small intestine. In combination with higher levels of oxidative stress during infection of SR-like communities, this suggests that modulation of biofilm or biofilm formation potentially contributes to the higher *V. cholerae* colonization level observed during infection of SS-bearing individuals.

### Microbiota Structure and Bile Salt Hydrolase Activity Modulates Bile Acid Composition and Affects *V. cholerae* Growth

**Figure 5:**
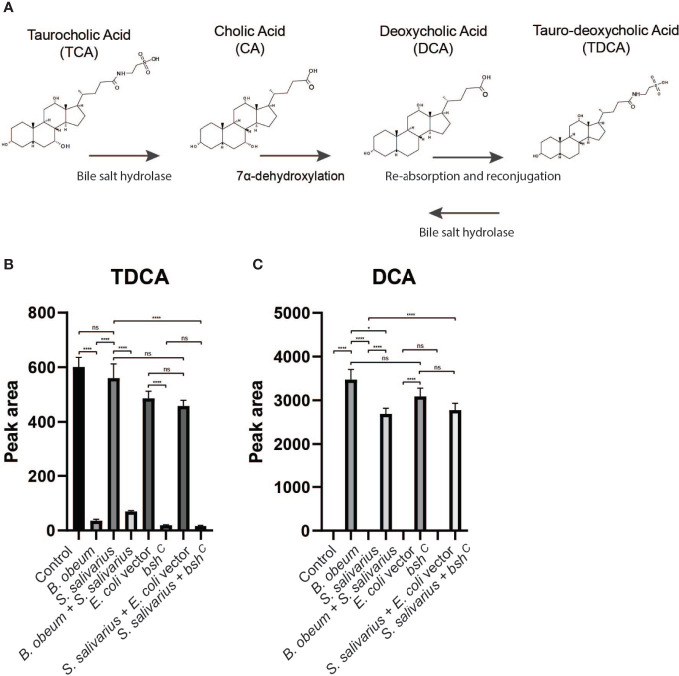
Bile salt hydrolase-dependent TDCA and DCA levels in infant mouse intestines. **(A)** Model of microbial modification of bile salt in the gut. **(B)** Tauro-deoxycholate (TDCA) and **(C)** deoxycholate (DCA) levels in intestinal homogenates after incubation with indicated strains. (n = 4, *t* test, *P < 0.05; ****P < 0.0001; ns, not significant).

**Figure 6:**
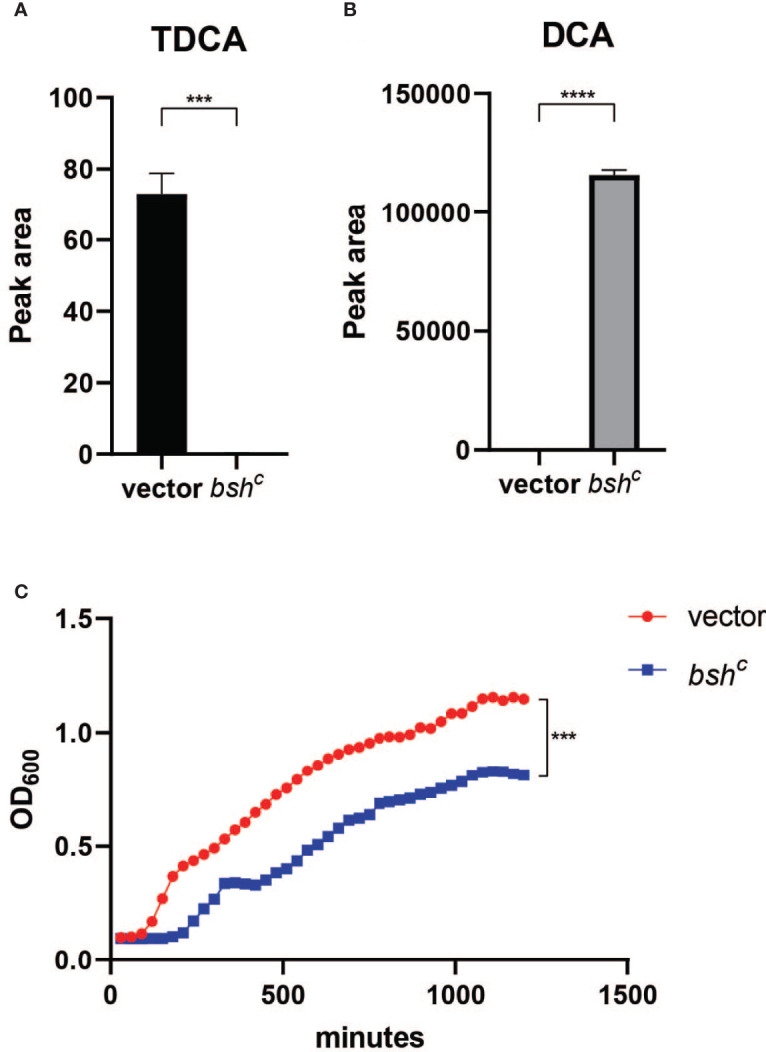
*Blautia obeum* bile salt hydrolase processes TDCA to DCA leading to inhibition of *V. cholerae* growth. **(A)** TDCA and **(B)** DCA levels after 24-h incubation of 2 mM TDCA with *E. coli* vector and *bsh^c^* strains as measured by mass spectrometry. **(C)** Effect on *V. cholerae* growth *in vitro* of addition of filtered supernatants from TDCA incubation with the indicated strains (n = 4, *t* test, ***P < 0.001; ****P < 0.0001).

Since different bile acids have been associated with inhibition of *V. cholerae* growth *in vitro* and regulation of biofilm production (Hay and Zhu, 2015), we examined how host bile might affect microbiota- and biofilm-dependent *V. cholerae* fitness during infection. Bile is a complex mixture secreted by the gallbladder into the proximal intestine in order to aid with the emulsification and absorption of dietary fats. A dominant component of bile are bile acids and their salt forms, the primary forms of which are synthesized in the liver from cholesterol, often in amino-acid conjugated forms. The cycle of bile secretion and subsequent reabsorption in the small intestine and the detergent effects of bile mean that commensals and pathogens that target this body site have evolved methods to detoxify bile components and also use them as signaling cues to time body site-specific gene expression programs. One key element of microbial bile response is the expression of bile salt hydrolase (BSH) enzymes, which catalyze the deconjugation of amino acids from primary bile, leading to a reduction in detergent activity and subsequent toxicity (Chand et al., 2017). Subsequent microbial action can also dehydroxylate the sterol backbone of bile acids, leading to a variety of different forms of bile acids that circulate within the intestine of animals housing resident microbes (De Smet et al., 1995). Taurocholic acid (TCA), an abundant amino-acid-conjugated bile species in mouse and human gut, is able to strongly activate the *V. cholerae* virulence regulatory cascade by promoting the formation of disulfide bonds in the upstream regulator TcpP (Yang et al., 2013). In contrast, the de-conjugated form, cholic acid (CA), and the deconjugated and dehydroxylated form deoxycholic acid (DCA) cannot strongly induce virulence. A previous study has shown that SR microbes and SS can help drive differential colonization resistance by modulating the level of TCA conjugation and virulence gene expression (Alavi et al., 2020); however, the interaction of *V. cholerae* with other TCA-derived bile acid metabolites has not been well described. This is especially important as DCA generated by BSH-dependent deconjugation of TCA is reabsorbed and reconjugated to taurine by the host, being secreted as taurodeoxycholic acid (TDCA) (**Figure 5A**). We therefore used mass spectrometry to analyze the bile salt composition in the intestinal homogenates, before and after incubation with *B. obeum* and *S. salivarius*. We found that TDCA signals in both control and SS are significantly higher than when incubated with *B. obeum* (**Figure 5B**). DCA levels in *B. obeum*-treated intestinal homogenates were several logs higher compared to *Streptococcus-*treated and control homogenates (**Figure 5C**). The accumulation of DCA suggests that *B. obeum* is able to de-conjugate TDCA to DCA in addition to de-conjugating TCA to CA. To examine whether the *B. obeum* BSH enzyme is able to catalyze this reaction, we examined TDCA and DCA levels in intestinal homogenates incubated with *E. coli* expressing *B. obeum* BSH (*bsh^c^*) compared to isogenic vector strains lacking any endogenous BSH activity. We observed the same low TDCA and high DCA signal patterns compared to vector control (**Figure 5**). To confirm the conversion from TDCA to DCA *via B. obeum* BSH, pure solutions of TDCA were added to *bsh^c^* cell pellets in PBS and incubated for 24 h. The supernatant was collected, filter sterilized, and used for mass spectrometry analysis. Consistent with our previous findings, the level of TDCA remaining was significantly higher in the vector control compared with *bsh^c^*, while DCA levels were ~600-fold higher in *bsh^c^* compared to vector control (**Figures 6A, B**). Previous studies have shown that DCA is inhibitory to *V. cholerae* growth (Hung et al., 2006). To confirm this, TDCA treated with *bsh^C^* or vector control was added to fresh *V. cholerae* culture and OD_600_ was taken every 30 min for 20 h. We observed that *V. cholerae* growth was inhibited by *bsh^c^*-processed supernatant compared to the vector strain(**Figure 6C**). Importantly, the presence of *B. obeum bsh* activity is able to provide inhibitory activity in the presence of *Streptococcus*, supporting the idea that the presence of infection-resistant microbes are able to restore colonization resistance to otherwise susceptible microbiota configurations (Alavi et al., 2020). Taken together, these results indicate that *B. obeum* is capable of converting TDCA to DCA *via* BSH expression, leading to *V. cholerae* growth inhibition during colonization.

### Biofilm-Dependent Bile Acid Resistance

**Figure 7:**
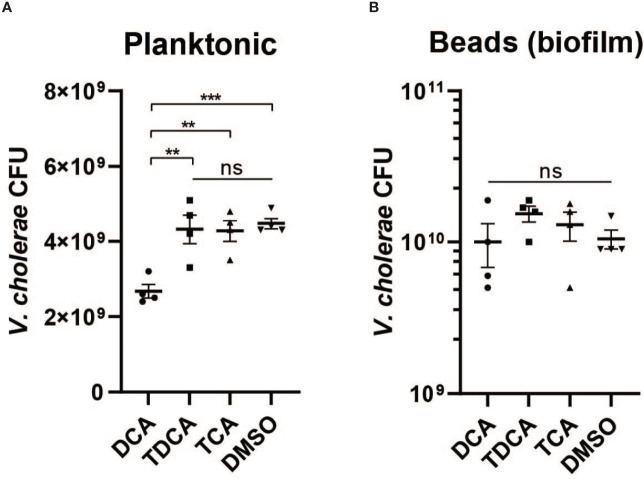
Biofilm provides *V. cholerae* resistance against secondary bile acid deoxycholate. Survival of **(A)** planktonic and **(B)** biofilm associated *V. cholerae* after 4 h of incubation with 1 mM of different bile acids including conjugated bile acids taurocholate (TCA) and taurodeoxycholate (TDCA), and the deconjugated secondary bile acid deoxycholate (DCA) (n = 4, T-test, **P < 0.01, ***P < 0.001; ns, not significant).

Previous studies have indicated that crude bile is able to induce biofilm formation, and that biofilm-associated cells are more resistant to bile toxicity (Hung et al., 2006). To test whether biofilm will contribute to *V. cholerae* resistance against different microbially modified bile acids, we first generated biofilm *V. cholerae* populations in media with beads as a surface for microbial attachment and subsequent biofilm development. We then exposed planktonic and biofilm-formed populations with products of microbial TCA metabolism: TCA, DCA, and TDCA. We found that TCA and TDCA do not significantly affect *V. cholerae* planktonic cell fitness in comparison with no bile acid (**Figure 7A**), but that after 4 h of treatment with 1 mM of DCA, planktonic *V. cholerae* exhibited a significantly lower survival. In contrast, biofilm-associated *V. cholerae* were insensitive to all bile treatments (**Figure 7B**). These repeatable data suggest that the formation of biofilm provides *V. cholerae* resistance toward certain bile acids present in the intestinal tract.

## Discussion

Increasing evidence demonstrates that the composition of the gut microbiota influences host defenses against pathogens (Ribet and Cossart, 2015; Cho et al., 2021). This study demonstrates that commensal-dependent manipulation of both *V. cholerae* and host behavior during infection leads to differential outcomes in *V. cholerae* fitness. *V. cholerae* in the context of infection-susceptible communities expresses more biofilm-associated genes, which protects the pathogen against the inhibitory activity of host oxidative mechanisms and inhibitory bile metabolites. The commensal community present during infection in turn affects both mechanisms; SR-colonized mice during infection express significantly higher levels of ROS/RNS production enzymes and display elevated hydrogen peroxide production compared to SS-colonized animals, and we show that one bile acid-processing enzyme within the key SR community member *B. obeum* can accumulate deoxycholate, which is inhibitory to planktonic, but not biofilm-associated, *V. cholerae*. These results demonstrate the multifactorial nature of commensal-associated colonization resistance; prior studies show that the dysbiotic, high *Streptococcus* microbiota is more susceptible to *V. cholerae* infection due to the inability to ablate bile-dependent virulence gene activation compared to microbiota containing *B. obeum* (Alavi et al., 2020).

The exact mechanism by which commensal microbes cause differential regulation of *V. cholerae* is not well understood. *V. cholerae* biofilm production *in vitro* is modulated by several pathways, including quorum sensing and bile response (Hung et al., 2006; Hay and Zhu, 2015). Quorum sensing (QS) plays a key role in the regulation of multiple infection-associated pathways in *V. cholerae* including biofilm production. The sensing of autoinducers leads to a series of dephosphorylation events leading to the repression of the expression of the small RNA regulators Qrr 1–4 (quorum regulatory RNAs) (Lenz et al., 2004). The absence of Qrr sRNAs allows for the expression of the master QS regulator *hapR*, which leads to the repression of the virulence genes and biofilm formation (Miller et al., 2002; Zhu et al., 2002; Hammer and Bassler, 2003; Shao and Bassler, 2014). HapR represses biofilm formation by binding to the regulatory regions of positive regulator *vpsT* and polysaccharide biosynthesis glycosyltransferase *vpsL*, also the first gene of the *vps*-II cluster in *V. cholerae* (Waters et al., 2008). Interestingly, production of the interspecies capable autoinducer AI-2 by *B. obeum* has been associated with downregulation of *V. cholerae* virulence and biofilm formation through HapR by transcription regulator VqmA (Liu et al., 2006; Hsiao et al., 2014; Papenfort et al., 2015). While the bi-association of *B. obeum* and *V. cholerae* in adult germ-free mice revealed no specific transcriptional signatures for biofilm gene regulation in fecal samples, our results suggest significant differences in biofilm regulation in the small intestine vs. large intestine (**Figures 4C, D**); compartment-specific microbiota effects on transcription may thus be masked in fecal transcriptomic data. Treatment with hydrogen peroxide *in vitro* also caused no significant increases in VPS gene expression, further suggesting that the regulation of biofilm is a host environment-specific phenotype (**Figure 4E**). *In vitro* studies have also examined the role of bile salts/acids in biofilm regulation. Hung et al. showed that deoxycholate is able to significantly induce *V. cholerae* biofilm formation in culture (Hung et al., 2006). The presence of bile also has been shown to alter VqmA activity and effect on *V. cholerae* virulence regulation (Mashruwala and Bassler, 2020). While this suggests that the SR community, with the ability to accumulate DCA *via* the activity of *B. obeum* BSH, should promote biofilm formation, the intersection of other signaling and regulatory pathways such as quorum sensing (Mashruwala and Bassler, 2020), and host oxidative stresses, which lead to increased biofilm formation (Wang et al., 2018), may combine to yield gut compartment- and microbiota-specific effects on *V. cholerae* biofilm regulation.

The role of microbial enzymes in manipulating the host bile acid pool is complex. TCA, CA, TDCA, and DCA are all metabolic products driven by microbial action; in germ-free animals, the bile acid pool is almost exclusively amino acid-conjugated primary bile molecules such as TCA (Sayin et al., 2013). Microbial BSH activity deconjugates primary molecules such as TCA, generating CA. Further microbial enzymatic action leads to dehydroxylation of CA to DCA (Molinaro et al., 2018), which can then be reabsorbed by the host, re-conjugated to amino acids, and re-secreted as TDCA (**Figure 5A**). Thus, multiple commensal pathways can contribute to highly community- and individual-specific bile acid pools. Microbial communities may interact to drive bile acid compositions *in vivo*; both Bacteroides and *C. scindens* within the SR community have been shown to exhibit both BSH and dehydroxylation activity (Kawamoto et al., 1989; Kang et al., 2008). Further complexity is added by the substrate specificity of microbial BSH, which has been grouped into eight phylotypes based on amino acid sequence similarity and substrate specificity (Song et al., 2019). Previous work by Alavi et al. showed that *B. obeum* BSH activity leads to restriction of *V. cholerae* infection by processing TCA, which is a potent activator of key *V. cholerae* virulence genes. In this study, we show that *B. obeum* is also able to process TDCA to DCA; accumulation of DCA leads to inhibition of planktonic, but not biofilm-associated, *V. cholerae*. The reduction of the *V. cholerae vps* gene expression in SR communities thus reinforces the colonization-resistant nature of host intestines bearing SR microbes.

This suggests that infection-resistant commensal communities, including *B. obeum*, can control *V. cholerae* fitness during infection by multiple, reinforcing mechanisms. Production of quorum sensing autoinducers and depletion of conjugated bile acids serving as virulence regulators interrupt the finely calibrated sequence of virulence gene expression regulatory events during early infection, coincident with an increased host oxidative ROS/RNS response compared to the high *Streptococcus* dysbiotic and permissive microbiota. While the precise mechanism leading to lower *vps* gene expression in colonization-resistant communities requires additional work to elucidate, the reduced biofilm formation leads to increased susceptibility to the heightened oxidative stress encountered by *V. cholerae* during infection in these microbiota contexts. Furthermore, *B. obeum* bile salt hydrolase activity not only depletes virulence-gene activating signals such as TCA but leads to the accumulation of DCA, the growth-inhibitory effects of which are accentuated by reduced *V. cholerae* biofilm production.

## Data Availability Statement

The datasets presented in this study can be found in online repositories. The names of the repository/repositories and accession number(s) can be found as follows: https://www.ncbi.nlm.nih.gov/bioproject/, 788969.

## Ethics Statement

The animal studies were reviewed and approved by the Institutional Animal Care and Use Committee, University of California, Riverside.

## Author Contributions

AH and JC conceived the study. JC performed the experiments. RL performed the RNA-Seq analysis. All authors contributed to the article and approved the submitted version.

## Funding

This study was supported by NIH grants GM124724 and AI157106 (to AH).

## Conflict of Interest

The authors declare that the research was conducted in the absence of any commercial or financial relationships that could be construed as a potential conflict of interest.

## Publisher’s Note

All claims expressed in this article are solely those of the authors and do not necessarily represent those of their affiliated organizations, or those of the publisher, the editors and the reviewers. Any product that may be evaluated in this article, or claim that may be made by its manufacturer, is not guaranteed or endorsed by the publisher.
